# The role of the occupational therapist in treatment of patients with prescription medicine dependence

**DOI:** 10.1192/j.eurpsy.2024.817

**Published:** 2024-08-27

**Authors:** D. Iskendri, L. Šťastná

**Affiliations:** ^1^Department of Addictology, Charles University, First Faculty of Medicine in Prague, Prague; ^2^University of West Bohemia, Faculty of Health Care Studies, Pilsen, Czech Republic

## Abstract

**Introduction:**

The abuse of prescription drugs (especially sedatives, hypnotics and opioid analgesics) is a serious and increasingly common phenomenon occurring across addiction clinics. Medications are prescribed for the treatment of chronic pain, sleep difficulties or as mood stabilisers in response to the rush of time and demands of performance. The onset of addiction is often protracted and subtle, but has a major impact on the quality of life and the health, economic or social status of the user. Patients may experience, among other things, cognitive impairment, fatigue, sleep disturbances, irritability, loss of motivation, headaches or impaired coordination of movements. This study is focused on cognitive impairment due to prescription drug dependence and how this impairment affects patients in everyday life.

**Objectives:**

This poster aims to introduce the audience to the possibilities of occupational therapy intervention in the context of addiction medicine.

**Methods:**

Data will be taken using standardized tests and questionnaires dealing with cognitive function. It will be conducted upon the patient’s admission to addiction treatment and again after six months of cognitive rehabilitation following the initial survey. Data are collected at the General University Hospital in Prague, Department of Addictology, Prague, Czech Republic.

**Results:**

Data are being collected.

**Conclusions:**

The case study manifests multidisciplinary approach in care of patients addicted of prescription medicine. The aim is a comprehensive view of all aspects of the patient’s life affected by prescription drug abuse with cognitive impairment.

**Grant affiliation:** This paper was made possible by the institutional support programme Cooperatio, research area Health Sciences and Grant No. 260632 within the Specific Academic Research.

**Disclosure of Interest:**

None Declared

